# Decarbonizing the global steel industry in a resource-constrained future—a systems perspective

**DOI:** 10.1098/rsta.2023.0233

**Published:** 2024-11-04

**Authors:** Takuma Watari, Benjamin McLellan

**Affiliations:** ^1^ Material Cycles Division, National Institute for Environmental Studies, Tsukuba, Japan; ^2^ Department of Industrial Ecology, Institute of Environmental Sciences, Leiden University, Leiden, The Netherlands; ^3^ Department of Socio-Environmental Energy Science, Graduate School of Energy Science, Kyoto University, Kyoto, Japan

**Keywords:** resource efficiency, demand reduction, circular economy, material flow analysis

## Abstract

Decarbonizing the global steel industry hinges on three key limited resources: geological carbon storage, zero-emission electricity and end-of-life scrap. Existing system analysis calls for an accelerated expansion of the supply of these resources to meet the assumed ever-increasing steel demand. In this study, we propose a different view on how to decarbonize the global steel industry, based on the principle that resource supply can only expand in line with historical trends and actual construction plans. Our analysis shows that global steel production cannot grow any further within a Paris-compatible carbon budget, resulting in a shortfall of approximately 30% against 2050 demand. This trajectory involves the phasing out of blast furnaces, along with strong growth in scrap recycling and hydrogen-based production. These findings highlight critical yet often overlooked challenges: (i) reducing excess demand while providing essential services, (ii) producing high-grade steel through upcycling scrap, and (iii) ensuring an equitable distribution of limited production across the globe. These perspectives contrast with those of the current agenda, which largely emphasizes the need to invest in new production technologies. Grounded in a physical basis, this analysis offers a complementary perspective for a more balanced debate in policymaking and industrial strategy.

This article is part of the discussion meeting issue ‘Sustainable metals: science and systems’.

## 1. Introduction

The functioning of modern society is dependent on steel in the form of products and infrastructure. Bridges, houses, cars and electronic devices all cannot function without versatile and affordable steel. Yet, the way we currently produce steel faces the fundamental challenge of ever-increasing greenhouse gas (GHG) emissions. Over the past two decades, GHG emissions from global iron and steel production have more than doubled, now accounting for approximately 9% of total emissions [1]. Clearly, the steel industry must transform itself in the fight against climate change. The question is: What kind of transformation is needed?

Existing studies to help us find answers in this domain can be divided into two main types. The first is based on economic modelling, which explores the 'cost-optimal' technology mixes and investment required to meet a given future steel demand within emissions constraints [2]. While this approach provides useful insights into the required investment scale, it often does not reflect the physical linkages in the series of steel cycles that include manufacturing, in-use stocks and waste management [3]. This, combined with the complexity of the models, often makes key assumptions, including those related to resource implications, opaque.

This challenge has been addressed by physical modelling, the second type of existing study, which traces the entire steel cycle while strictly adhering to the law of mass conservation [4]. The approach is straightforward, and the assumptions are transparent, making it easy to interpret the results [5]. However, existing scenario exploration in these two types of studies has been based exclusively on the principle of ‘forecasting demand and backcasting supply’, where we first forecast steel demand and then backcast supply configuration to meet that demand [6]. Because of the modelling structure, this approach heavily relies on certain essential resources, such as geological carbon storage [7]. As a result, there has been less emphasis on the actions of actors who use steel products (e.g. manufacturers, urban planners and general consumers) [8].

This paper proposes a new approach based on the principle of ‘forecasting supply and backcasting demand’. The approach follows the principle that resource supply can only expand in line with historical trends and actual construction plans. We, therefore, first examine the resource implications of all possible transformations of the global steel industry. This then allows us to anticipate the scale and speed at which different production technologies could replace today’s production. The proposed approach highlights the magnitude of the challenge of living well with less steel production and provides key insights into how to decarbonize the global steel industry in a resource-constrained future.

## 2. Resource requirements of carbon reduction options

In the extensive history of research and consideration, existing studies have explored a wide spectrum of options for decarbonizing the steel industry. These include initiatives focusing on energy efficiency, scrap recycling, solid biomass, smelt reduction, carbon capture and storage (CCS), hydrogen-based reduction and electrolysis [9]. Within this spectrum, recent attention has focused primarily on three key pillars—CCS, hydrogen and recycling—as central components of the steel decarbonization effort [10].

CCS is typically applied to the blast furnace (BF) and basic oxygen furnace (BOF) production route, which dominates approximately 70% of total crude steel production [11]. With limited potential for further energy efficiency improvements in the BF-BOF route [12], there has been a consistent focus on deploying CCS technologies over the past decades [13]. Carbon capture itself is a relatively mature technology, encompassing various methods such as chemical absorption, physical adsorption, membranes, calcium looping, chemical looping and sorption-enhanced water gas shift [14]. These approaches are designed to selectively separate CO_2_ from other gases, enabling its subsequent compression and underground storage. However, the critical challenge lies in the storage phase, where ensuring extensive and reliable geological storage of the captured carbon remains a key constraint [15]. Inadequate management of this phase could potentially lead to the release of stored CO_2_ into the atmosphere, contamination of groundwater, damage to ecosystems and induced seismicity [16]. In many cases, building public acceptance for CCS projects has been a daunting task due to concerns about these risks [17]. The efficacy of this option thus hinges on the availability of extensive and reliable geological storage for the captured carbon, with approximately 2 tonnes of carbon storage required for every tonne of steel produced via the BF-BOF route.

Hydrogen-based reduction of iron ore has gained increasing traction in recent years, particularly with the recent resurgence in interest in hydrogen as an energy carrier [18]. This option involves the use of hydrogen gas as a reducing agent, where the hydrogen reacts with iron oxide ore to remove oxygen from the ore without emitting CO_2_ (at least directly) [19]. While several technologies have been explored for variations of ore quality, energy source and desired steel grade, the principal is to use hydrogen gas as a reducing agent in a shaft furnace to convert iron ore pellets to direct reduced iron (DRI) [20]—a process that is currently undertaken with natural gas. The DRI produced is further refined in an electric arc furnace (EAF) to yield steel. It is important to note, however, that this option is not resource-free either. To achieve zero emissions, the process requires green hydrogen produced by electrolysis of water, which in turn requires a significant amount of zero-emission electricity [21]. The system-wide electricity requirement for the H_2_ DRI-EAF route (i.e. including water electrolysis) is estimated at roughly more than 10 times that of the BF-BOF route or 7 times that of the scrap-based EAF route [22]. Alternatively, hydrogen produced from fossil fuels with CCS could be used, further exacerbating the requirement for carbon storage. Thus, careful consideration of the implications of hydrogen-based reduction on electricity demand is essential to avoid over-promising and under-delivering.

Scrap recycling has long been practised in industry for its economic competitiveness and, more recently, its alignment with circular economy principles and climate change mitigation [23]. By utilizing EAF for scrap recycling, the industry can produce almost zero-emission steel when operated with zero-emission electricity and at a lower cost than alternative methods [24]. The process only requires modest zero-emission electricity without the need for extensive carbon storage to achieve zero emissions. However, the expansion of this production method can be physically constrained by the availability of scrap, particularly end-of-life scrap [25]. The logic is straightforward: expanding scrap-EAF production necessitates a greater supply of scrap. Two critical factors come into play in this realm: the average lifespan of steel products and the recovery rate of end-of-life scrap. While the lifespan of steel-containing products varies considerably, ranging from a few years for electrical equipment to several decades for infrastructure, a weighted average across all products gives an approximate lifespan of 38 years [26]. The maximum recoverable amount of end-of-life scrap in the coming decades is thus constrained by the long lifespan of steel products. Another key consideration is the recovery rate of end-of-life scrap, which has typically been in the 50–90% range globally over the last decade [27]. This suggests that there is limited room for further improvement, as pushing it to close to 100% would be energy-intensive and impractical due to the highly diluted nature of scrap distribution [28]. Consequently, the expansion of the scrap-EAF routes is not unlimited but hinges on a stable supply of end-of-life scrap.

Taken together, the production of zero-emission steel depends on three key limited resources: geological carbon storage, zero-emission electricity and end-of-life scrap. The scale of the uptake of these three resources could therefore determine the scale of the feasible supply of zero-emission steel. This theory forms the basis of the ‘forecasting supply and backcasting demand’ approach proposed in this study, as opposed to ‘forecasting demand and backcasting supply’.

## 3. Forecasting demand and backcasting supply

The approach of ‘forecasting demand and backcasting supply’ is adopted by almost all the existing studies, where we first forecast steel demand and then backcast supply configuration to meet that demand within emissions constraints. This is usually done by solving a cost minimization problem [29], a typical outcome of which is a series of reports by the International Energy Agency (IEA) [2]. To illustrate the difference between the results of the different approaches, we take the future steel supply-demand scenario from the IEA report as a prime example of the result of the ‘forecasting demand and backcasting supply’ approach [30].

This future steel supply-demand scenario is translated into resource requirements in the following way: first, carbon storage requirements are calculated as the difference between carbon emissions from steel production and the carbon budget. The carbon budget is based on limiting the global mean temperature increase to 1.5°C with a 50% probability, in line with the Paris Agreement commitments [31]. We allocate this Paris-compatible carbon budget for global steel production by multiplying the steel industry’s current emissions by the required annual emissions reduction rate [32]. This reflects the assumption that the global steel industry contributes to mitigation pathways in proportion to other sectors. Second, electricity requirements are calculated by simply multiplying each unit of steel production by an electricity requirement per tonne of steel produced. In this case, electricity requirements for the H_2_ DRI-EAF process include electricity use for green hydrogen production with an electrolyser efficiency of 45 kWh kg^−1^ H_2_ and a hydrogen mass flow rate of 1.5 [33] (i.e. 50% oversupply of hydrogen for full conversion of iron ore in the shaft). Third, scrap requirements are estimated on the basis of physical mass balancing. Specifically, a time cohort model, which estimates how steel-containing products are disposed of over time, enables this calculation [34]. The future steel supply-demand scenarios and resource requirements obtained by these steps are interpreted as benchmarks for comparison with the results obtained by the proposed new approach.

## 4. Forecasting supply and backcasting demand

Our proposed approach starts with a plausible supply of key resources—geological carbon storage, zero-emission electricity and end-of-life scrap—and then estimates the maximum steel production that can be achieved within these resource constraints. The estimated steel production ultimately determines the allowable steel demand. This is the opposite of the conventional approach, which starts with an assumption of future steel demand. Grounded in physical mass balancing equations connected with an optimization routine, the model explicitly considers the supply of key resources, which follows the principle that resource supply can only expand in line with historical trends and actual construction plans. Detailed mathematical representations and data can be found in the electronic supplementary material, with the basic concept of this approach developed in our previous research [22]. The major difference between this work and previous work is the redefinition of the physical basis with empirically grounded data and the comparison with the conventional approach. We believe that this exercise offers a complementary view on how to decarbonize the global steel industry in a resource-constrained future and highlights critical yet often overlooked challenges.

**Figure 1.**
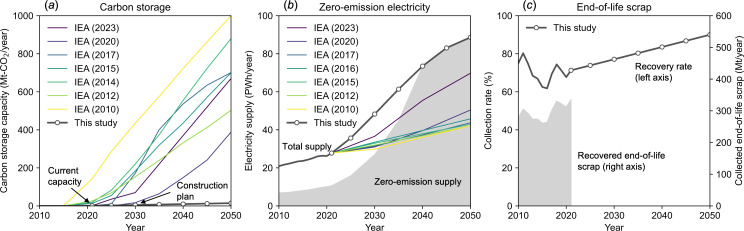
Assumptions regarding the future supply of three key resources. (*a*) Carbon storage. (*b*) Zero-emission electricity. (*c*) End-of-life scrap.

In this model, the future steel demand is not pre-determined but the uptake of three essential resources must be. The following assumptions are made in this space. First, the potential uptake of geological carbon storage is based on the actual construction plans. CCS in the steel sector has not progressed as per the IEA scenarios for decades; CCS deployment in the steel sector has always lagged behind the IEA scenarios [22]. Furthermore, there is a significant gap between the IEA scenarios and the actual construction plan for 2030 [35]. Given the history of failures and the magnitude of the challenges, a conservative assumption would be that CCS deployment will only proceed in line with the actual construction plan. We therefore assume the potential uptake of carbon storage independently of the IEA scenarios by extrapolating current operational capacity and 2030 construction plans up to 2050 (figure 1*a*).

Second, the potential uptake of zero-emission electricity is based on historical growth data, as the story for this resource is significantly different from that for carbon storage. An earlier study argues that energy technologies can be roughly divided into two groups based on their rates of improvement [36]. The first group includes technologies where inflation-adjusted costs have remained broadly constant over the period and deployment has stagnated; CCS falls into this group. In contrast, the second group has seen a dramatic increase in deployment while costs have fallen almost exponentially; renewable electricity technologies belong to this group. Historically, most energy system models, including the IEA’s model, have consistently underestimated deployment rates for renewable energy technologies and overestimated their costs [37]. Based on this empirical evidence, we assume that zero-emission electricity grows at rates consistent with historical data (i.e. the Faster Transition scenario in [36]), resulting in a faster transition to renewable and electrified energy systems than the historical IEA estimates (figure 1*b*). As electricity demand is expected to increase in all sectors [38], the electricity available to the steel sector is assumed to increase in proportion to the total electricity supply.

Third, the potential uptake of end-of-life scrap is based on the best practices in certain regions. Over recent decades, global recovery rates of end-of-life scrap have generally been in the range of 50–90%, depending on the product category [27]. Some developed countries have achieved average recovery rates approaching 90% [39]. However, bringing this value closer to 100% is impractical due to the highly diluted nature of scrap distribution [28]. We thus expect global average scrap recovery rates to approach 90% in the coming decades, in line with economic developments (figure 1*c*). It is important to note that the absolute amount of scrap recovered is determined within the model using mass balance equations. Therefore, what is given externally to the model is the scrap recovery rate and not the absolute amount of scrap recovered.

Obviously, predicting the likely rate of change is rather complex, involving myriad logistic, economic, social, and political factors. For instance, it is possible that the steel industry could build up more electricity supply or use excess electricity from variable renewables in a way that does not necessarily take from other sectors [40]. The investment in such captive energy supply, while possible, is not assumed to reflect actual deployment. Our intention here is to make assumptions based as much as possible on empirically grounded data in a transparent way. Results should always be interpreted alongside the assumed resource requirements, and we are careful to make this easy to do in the figures and text.

## 5. Different decarbonization pathways

Comparing future scenarios from the two approaches brings different insights into what interventions we should focus on. In both scenarios, carbon emissions from global steel production follow a mitigation path based on a Paris-compatible carbon budget. The difference lies in whether future demand is predetermined or future supply is predetermined. This simple comparative analysis shows that both scenarios stay within the carbon budget but require significantly different types and levels of steel production and resource uptake.

**Figure 2.**
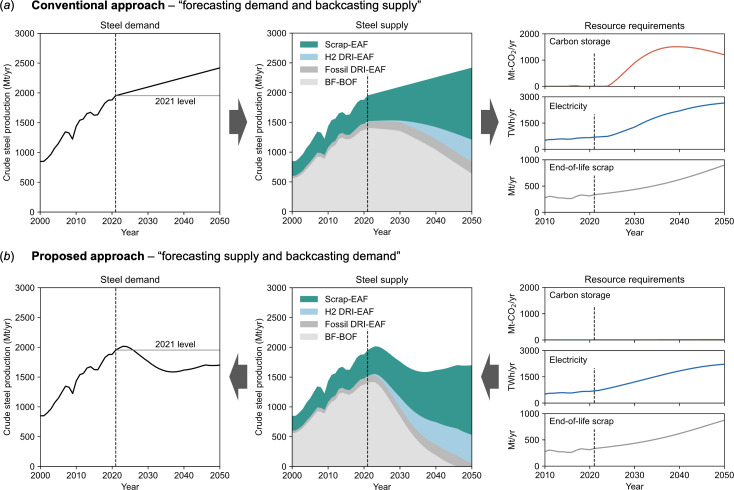
Global steel demand, supply and associated resource requirements to stay within a Paris-compatible carbon budget by 2050. (*a*) Conventional approach: forecasting demand and backcasting supply. (*b*) Proposed approach: forecasting supply and backcasting demand.

Under the ‘forecasting demand’ approach (figure 2*a*), total steel production can continue to grow until 2050 but will require a zero-emission electricity supply equivalent to four times the current total electricity demand of the global steel industry. More notably, the required level of geological carbon storage in 2050 will be approximately 1000 times the current capacity in the steel sector. Despite the expansion of scrap-EAF production, the main production routes remain the ore-based BF-BOF process. This is because the maximum scrap supply is defined by historical production levels. As long as total production continues to increase without a declining rate of acceleration, the scrap supply will always be less than total production. The main implication of this scenario is that the key resource supply needs to be accelerated on an unprecedented scale.

In contrast, the scenario generated by the ‘forecasting supply’ approach only requires resource supply in line with historical trends and actual construction plans (figure 2*b*). The consequence of this is a reduction of approximately 30% in total steel production by 2050 compared to the business-as-usual scenario. A closer look at the production routes reveals three key trends. First, BF-BOF production will be rapidly phased out due to limited carbon storage capacity. Second, H_2_ DRI-EAF production will replace some of the decline in BF-BOF production and grow to a level equivalent to about a quarter of current total production. Third, scrap-EAF production will triple from current levels by 2050, accounting for the largest share of production. The growth in EAF steel production, either using scrap or hydrogen-reduced iron, will be sufficient to compensate for the decline in BF-BOF production but will not allow total production to grow.

The main implication of this scenario contrasts with that obtained through the conventional ‘forecasting demand’ approach—we need to focus more on using less steel while still meeting our service needs, including shelter, mobility and communication. Specifically, decarbonizing the global steel industry in a resource-constrained future will require meeting the service needs of the world’s population with approximately 30% less steel than the business-as-usual scenario.

## 6. Challenges in a recycling-dependent world

Another key picture in the scenario generated by the ‘forecasting supply’ is the growing role of scrap-EAF in total production. Together with the demand reduction, the scenario requires about 70% of production to come from scrap-EAF by 2050. Such a shift could pose two major challenges: downcycling and international inequality.

**Figure 3.**
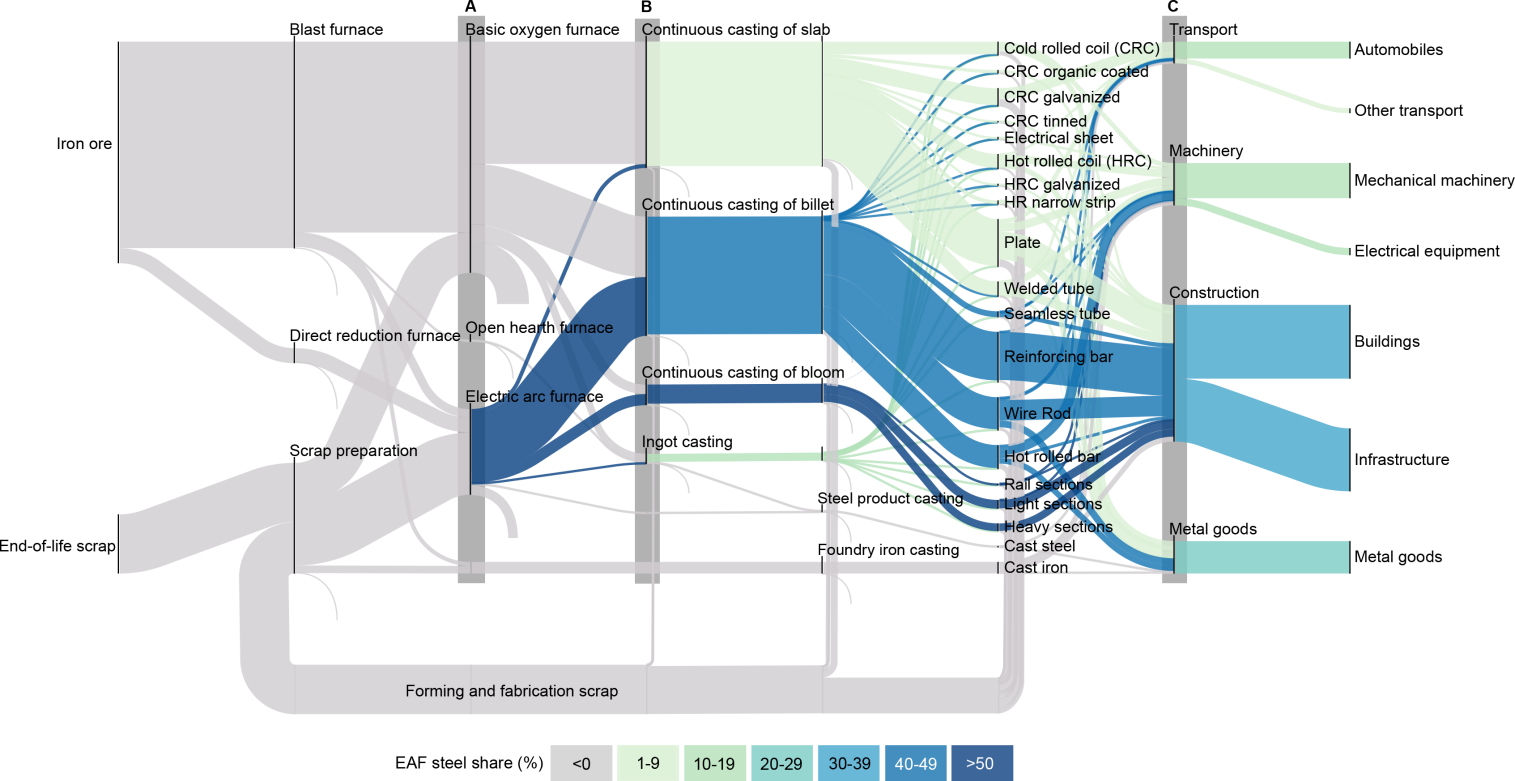
Global steel flows from raw materials to end-use products in 2020. The thickness of each flow reflects the weight of steel; the colour reflects the proportion of steel produced from EAF. The iron ore flow represents the iron embedded in the ore and excludes the mass of oxygen and gangue. The method is based on [43] with the same assumption for EAF steel processed into slabs with [44]. The diagram was designed with floWeaver software with all flows scaled to Mt yr^−1^ [45]. Three key flows are marked on this diagram: (A) Approximately 70% of steel is currently produced by the BF-BOF process and the remaining 30% by EAFs using either scrap or DRI as the primary feedstock. (B) Almost all EAF steel is currently processed into billets and blooms, with a limited capacity for slabs. (C) There is a marked difference between the share of EAF steel in finished steel products used in construction and in other industries.

Downcycling refers to the process of recycling materials into products of lower quality or value [41]. It occurs when materials are recycled or reprocessed in a way that leads to a degradation in their properties or performance compared to the original material. In the context of scrap steel recycling, downcycling generally refers to the process by which scrap steel is recycled into construction-related products (e.g. rebar) rather than into original relatively higher-value products (e.g. cold-rolled coil) [42]. To get an intuitive sense of the scale of this challenge, figure 3 shows a map illustrating how various steel products were produced and manufactured in 2020, using the same methodology as in the literature [43]. Three key flows are marked on this diagram, highlighting the major challenges of the coming decades:

— Approximately 70% of steel is currently produced by the BF-BOF process and the remaining 30% by EAFs using either scrap or DRI as the primary feedstock. EAF capacity will need to be scaled up to fully capture the future scrap flow.
— Almost all EAF steel is currently processed into billets and blooms, with a limited capacity for slabs. The casting equipment at EAF facilities will need to be updated so that slabs can also be made.
— There is a marked difference between the share of EAF steel in finished steel products used in construction and in other industries. Technologies and systems will need to be put in place to control impurities in scrap steel and enable upcycling rather than downcycling.

Overcoming the second and third challenges can be particularly challenging because the impurities, mainly copper, mixed with the end-of-life scrap steel are difficult to remove in current steelmaking processes due to thermodynamic properties [44]. Higher levels of impurities in steel can lead to increased susceptibility to cracking at high temperatures and a degradation of steel quality, preventing the use of EAF steel in some products that require a high level of quality control [46]. For example, copper tolerances are much tighter for deep-drawn sheet steel (≤0.06 wt%Cu), which is mainly used in the automotive industry to make body panels, doors, hoods and fenders, than for reinforcing bar (≤0.4 wt%Cu), which is mainly used in the construction industry [47]. Therefore, simply being able to live well with less steel production is not enough to prepare for a resource-constrained future. There is an urgent need to find a way to produce high-quality steel products from scrap-EAF production.

**Figure 4.**
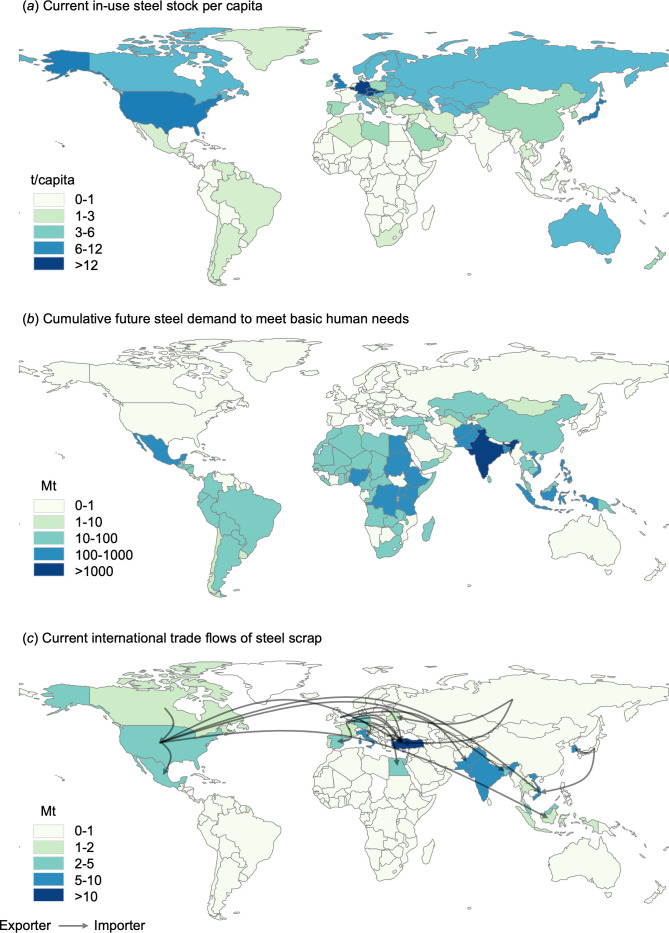
World maps illustrating key steel data. (*a*) In-use steel stock per capita in 2014. (*b*) Cumulative steel demand for meeting five essential services (i.e. electricity, water, sanitation, shelter and mobility) from 2015 to 2050. (*c*) International trade flows of steel scrap in 2020. Please note that the cumulative steel demand shown here is a net addition to stocks, i.e. it does not include material demand in countries that have already accumulated enough steel stocks to meet basic human needs. Only international trade flows of more than 1 Mt are shown. Data for (*a*) and (*b*) are taken from [48], while data for (*c*) are taken from [49].

Furthermore, the issue of international inequality could intensify if scrap-based EAF production becomes the dominant mode of steel production. The reason for this is simple: the future availability of scrap-based EAFs depends on the physical availability of end-of-life scrap, which can vary widely from region to region. There is currently a notable trend in this regard: countries in the Global North have accumulated significant amounts of steel stocks, averaging around 10 tonnes per capita, while countries in the Global South are still in the early stages of stockpiling, with less than 1 tonne per capita (figure 4*a*). This trend has significant implications for global equity, as it essentially means that only a limited number of countries in the Global North can monopolize the benefits of producing EAF steel.

To complicate matters further, the majority of steel demand over the next decade is likely to come from countries in the Global South to meet basic human needs, such as access to energy, water, sanitation, shelter and mobility (figure 4*b*). This highlights a stark discrepancy: it is the Global North that possesses valuable steel stocks that can be recycled in the future, yet it is the Global South that requires substantial quantities of steel to meet basic needs.

International trade has been expected to mitigate such inequalities in resource availability [50]. At the moment, more than 100 Mt of scrap steel is traded internationally, with the majority being exported from countries in the Global North, such as the USA, Japan and Germany (figure 4*c*). Major importers include Turkey, India and Vietnam where such scrap imports are essential for the domestic recycling industry. In theory, if properly managed, such international trade could play a vital role in mitigating international inequality in scrap availability and fostering an ‘inclusive circular economy’ [51]. Indeed, scrap generated in the Global North is currently helping to meet some of the demand for construction-grade steel (e.g. reinforcing bar) with a higher copper tolerance in the Global South [52]. However, there has been a global trend towards prioritizing securing valuable resources domestically, particularly in the context of critical materials [53]. If countries in the Global North also seek to monopolize their scrap steel domestically without considering equity concerns, countries in the Global South may lose the opportunity to meet their needs with zero-emission steel. The role of international trade in this context is undoubtedly complex, but these perspectives highlight the critical importance of addressing international inequality on the agenda. This is essential if decarbonization is to be achieved on a global scale, not just in a limited number of countries in the Global North.

## 7. Policy implications

Taken together, the ‘forecasting supply and backcasting demand’ approach proposed in this study highlights crucial but currently under-emphasized challenges in decarbonizing the global steel industry: (i) limited production, (ii) downcycling, and (iii) international inequity. These perspectives contrast with those of the conventional approach, which almost exclusively emphasizes the need to invest in new production technologies. Given the staggering scale of efforts required for decarbonizing the steel industry, this analysis offers a complementary perspective for a more balanced debate in policymaking and industrial strategy.

Firstly, to cope with limited production, the obvious debate that needs to be had is how to reduce future demand. Throughout the life cycle of products or infrastructure, we have a number of options to reduce steel demand while still providing the necessary services (i.e. shelter, mobility and communication) [54]. These strategies include eliminating overdesign [55] and right-sizing [56] at the design stage to reduce material purchase, improving specifications to minimize construction waste [57] and promoting shared and extended use through effective reuse and refurbishment to reduce new product demand [58]. The benchmark for these measures is a 30% reduction in steel demand by 2050 compared to business as usual, which is technically feasible given current knowledge [59]. However, the implementation of such measures may greatly depend on appropriate incentives and policy support, as there is currently little motivation to reduce either steel demand or supply [60]. Given the strong link between the mass of steel produced and profits in the steel industry, there is an urgent need to develop new industrial strategies that will allow continued profitability despite reduced mass output, together with adjustments to regulations, product standards and pricing mechanisms [61]. The analysis presented here provides a clear signal to open the debate on these issues and gives a benchmark for the action that needs to be taken.

Secondly, the management of downcycling depends greatly on how impurities are dealt with. The main cause of downcycling at the moment is tramp elements, mainly copper [62]. Copper is thermodynamically difficult to remove once it enters the steelmaking process [63]. We must therefore carefully control copper in steel recycling by, for example, a more careful dismantling of parts and better shredding and sorting, or by reducing the amount of copper in the product design in the first place [64]. As indicated in figure 3, only a handful of plants have the capacity to produce slabs from EAF steel, with the USA emerging as a notable example [65]. Strategies adopted by these facilities include dilution of impurities through virgin material blending, strategic proximity to sources of relatively clean scrap (e.g. car stamping plants), shorter oxidation periods and chemical composition adjustments during remelting [66]. Implementing these measures globally may require new technologies and systems, but the need for investment in these is often left out of the decarbonization agenda. This analysis once again provides evidence to open the debate on these areas to enable the upcycling of steel scrap.

Finally, and perhaps most importantly, there is an urgent need to put equity concerns at the heart of the decarbonization agenda. The existing disparity in steel use worldwide underscores a critical reality: the Global South is in the process of developing its steel stocks to enable a decent life for all, and this stage of development is inherently more carbon-intensive than later stages, in part due to the limited availability of scrap. Such a perspective is already discussed in an earlier paper [24], but our analysis with the perspective of limited total production reinforces its importance—what we need to do is to ensure an equitable distribution of this ‘limited’ production across the globe. This imperative underlines the need for a nuanced approach, taking into account the different stages of development and the challenges faced by different regions. Specific strategies could include financial and non-financial assistance, climate clubs and global treaties [67]. Yet, again, these perspectives are often left out of the decarbonization agenda. This analysis cannot provide specific solutions in this area, but it can be a stepping stone to start a detailed, region-by-region analysis to offer specific solutions.

Overall, the ‘forecasting supply and backcasting demand’ approach, as highlighted in this study, illuminates the multifaceted challenges of decarbonizing the steel industry. Engaging in a debate on issues of limited production, downcycling and international inequality provides an opportunity for policymakers and industry leaders to craft more comprehensive strategies that go beyond conventional supply-side technological solutions.

## 8. Future research avenues

As this paper draws to a close, this final section aims to discuss the avenues for future research. Our analysis essentially underscores three critical areas: reduction, upcycling and equity. We argue that future research agendas should prioritize the exploration of technological and system advances within these domains. For example, future research efforts could focus on the development of less steel-intensive alternative pathways for countries in the Global South, enabling them to deviate from the historical development paths of their counterparts in the Global North [68]. Technological advances that enable the upcycling of scrap steel, such as deep learning-based image analysis [69] or strategic oxygen control in melting furnaces [70], clearly need to be further explored. There is also an opportunity to explore the integration of conventional circular economy principles with an equity-focused perspective to enable a just transition [71].

Conceptually, the integration of the three perspectives—reduction, upcycling and equity—raises several key questions that warrant thorough investigation: How does the scrap monopoly in certain countries affect global material flows and carbon emissions? What are the optimal inter-country material flows (if any) when equity is at the heart of the debate? What benchmarks should each country achieve in terms of reduction and upcycling within an optimal paradigm of inter-country material flows? What business models and supporting policy measures can maintain viability for steel producers? We call for further research to address these questions in order to enable the timely and sufficient decarbonization of the global steel industry.

## Data Availability

The datasets supporting this article are uploaded as part of the electronic supplementary material [72].
